# Low-dose aspirin and burr-hole drainage of chronic subdural hematoma: study protocol for a randomized controlled study

**DOI:** 10.1186/s13063-018-3064-y

**Published:** 2019-01-21

**Authors:** Maria Kamenova, Christian Mueller, Michael Coslovsky, Raphael Guzman, Luigi Mariani, Jehuda Soleman

**Affiliations:** 1 Department of Neurosurgery, University Hospital of Basel, Spitalstrasse 21, 4053 CH Basel, Switzerland; 2 Department of Cardiology, University Hospital of Basel, Petersgraben 4, 4031 CH Basel, Switzerland; 3 Clinical Trial Unit University Hospital of Basel, Spitalstrasse 12, 4031 CH Basel, Switzerland

**Keywords:** Burr-hole drainage, acetylsalicylic acid, chronic subdural hematoma, blood thinners, neurosurgery

## Abstract

**Background:**

Low-dose acetylsalicylic acid (ASA) in patients with chronic subdural hematoma (cSDH) represents a significant neurosurgical challenge. While continuation of ASA during the perioperative phase might increase recurrence and bleeding rates, discontinuation increases the risk of thromboembolic events. The aim of this study is to compare the postoperative recurrence and cardiovascular complication rates of patients undergoing burr-hole trepanation for cSDH with and without discontinuation of ASA.

**Methods:**

In this prospective randomized, placebo-controlled, double-blinded study we include all patients undergoing burr-hole drainage of cSDH who are under ASA treatment. The patients are randomized into two groups, one receiving ASA and the other placebo perioperatively. The study primarily seeks to compare the rate of recurrent events under ASA to that under placebo treatment. Secondary objectives are thromboembolic event rate, perioperative blood loss, postoperative anemia, intra- and postoperative blood transfusion rate, and clinical outcome.

**Discussion:**

To date, there is no evidence-based consensus on how to manage patients undergoing burr-hole drainage for cSDH who are under ASA treatment. Therefore, the decision to maintain or interrupt ASA treatment is based mostly on the surgeons’ preference. A randomized placebo-controlled study for this frequent question is urgently needed in order to provide class I evidence for the best possible treatment of this large group of patients.

**Trial registration:**

ClinicalTrials.gov: NCT03120182. Initial Release: 19.04.2017. Study protocol: V2_23.02.2017

**Electronic supplementary material:**

The online version of this article (10.1186/s13063-018-3064-y) contains supplementary material, which is available to authorized users.

## Background

Acetylsalicylic acid (ASA) is a drug widely prescribed for the primary and secondary prevention of coronary artery disease [1, 2]. ASA reduces the risk of cardiovascular death or subsequent attacks in patients with previous myocardial infarction, unstable angina pectoris, stroke, or transient ischemic attacks [3, 4]. Nearly 40% of patients who undergo non-cardiac surgery worldwide have or are at risk of coronary artery disease; of these, 4% per year develop a major intraoperative cardiovascular complication, including cardiac death, non-fatal myocardial infarction and cardiac arrest [5, 6]. In-hospital mortality due to perioperative myocardial infarction ranges from 15% to 25% [6–8]. Moreover, perioperative myocardial injury (PMI) after non-cardiac surgery, defined as troponin increase of ≥ 14 ng/L, was associated with substantial short- and long-term mortality [9].

The incidence of chronic subdural hematoma (cSDH) is estimated at 1.7–18 per 100,000 people and rises to 58 per 100,000 in people above the age of 65 [10]. Due to the significantly higher prevalence among patients older than 65 years (69%), 41% of these patients are under platelet aggregation inhibitor or oral anticoagulant treatment [11]. Antiplatelet therapy in patients with cSDH presents a significant neurosurgical challenge. Although patients seem to be at greater risk for cSDH while taking these medications, it remains unclear how antiplatelet therapy affects recurrence rates [10, 12–14]. Furthermore, in balancing the increased cardiovascular risk with increasing prevalence of cSDH, a lack of guidelines and recommendations persists regarding the perioperative management of patients with antiplatelet therapy. Some retrospective studies showed a trend for higher recurrence rates when ASA is continued in the perioperative phase after burr-hole drainage, without reaching significance [15–17]. Most surgeons still prefer to discontinue and/or revert antiplatelet therapy prior to surgery since they fear the risk of recurrence or intracranial bleeding [18, 19]. Although studies evaluating the bleeding risks once ASA is continued during the perioperative period exist (e.g., POISE, PEP trial, STRATAGEM) [6, 20–22], they exclude neurosurgical patients and are therefore not relevant with regards to neurosurgical patients. The effect of continuous ASA treatment after burr-hole drainage of cSDH remains uncertain and therefore a prospective randomized study elucidating the effect of ASA continuation on the recurrence rate and of discontinuation on the thrombotic events rate is imperative.

## Methods

### Aim

The aim of this study is to undertake a randomized controlled trial on the recurrence and cardiovascular complication rates of patients undergoing burr-hole drainage of cSDH with and without discontinuation of low-dose ASA in the perioperative phase.

### Study design

**Fig. 1:**
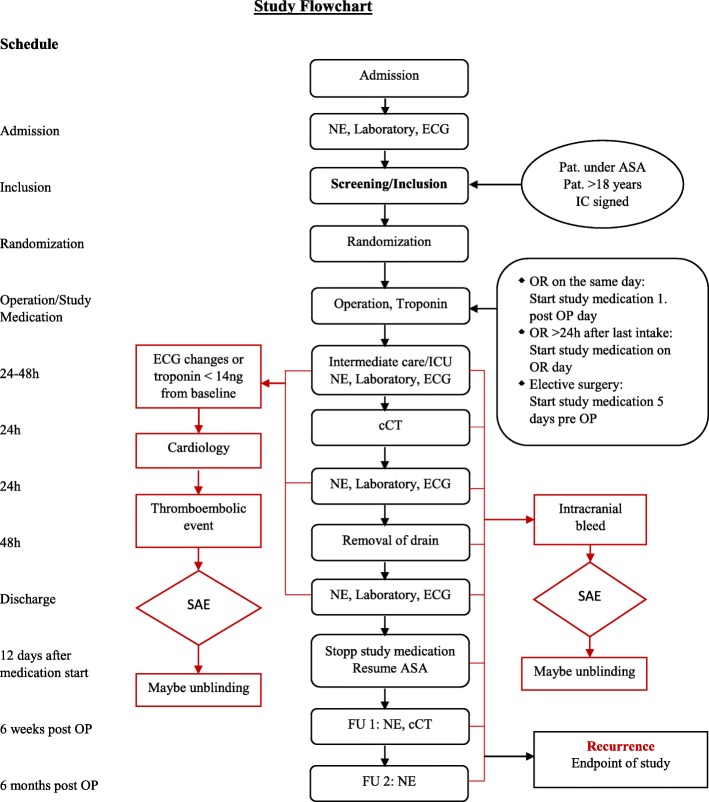
Study flow chart

This will be a prospective, randomized, placebo-controlled, double-blinded superiority trial performed at the Neurosurgical Department of the University Hospital of Basel (Fig. 1 and Additional file 1).

### Outcomes

#### Primary study outcome measures

The primary study outcome measure is recurrence of cSDH requiring revision surgery within 6 months.

#### Secondary study outcome measures

Secondary outcome measures include the following:

- Thrombotic events, namely ST-segment elevation myocardial infarction/non-ST-elevation myocardial infarction/asymptomatic PMI (defined as troponin increase of ≥ 14 ng/L compared to baseline troponin levels), cerebral stroke, peripheral arterial occlusion
- Other bleeding events apart from recurrent cSDH, namely acute subdural hematoma, acute epidural hematoma, intraparenchymal bleeding or subarachnoid hemorrhage managed operatively or conservatively
- Intraoperative blood loss
- Amount of blood/fluid collected in the drain
- Postoperative anemia (hemoglobin < 80 mg/L)
- Operation time (skin-to-skin)
- Hospitalization time
- Intra- and postoperative blood transfusion rate
- Clinical outcome (modified Rankin scale (mRS), Glasgow Outcome Scale (GOS), Markwalder score)

### Inclusion criteria

- Informed consent as documented by signature
- Patients undergoing burr-hole drainage for cSDH who are under low-dose ASA treatment (Aspirin cardio^®^ 100 mg once a day)
- ASA users under discontinued ASA treatment (e.g., conservatively managed for a period of time and ASA was discontinued)
- Patients older than 18 years

### Exclusion criteria

- Patients under the age of 18 years
- A recent (30 days before randomization) major cardiac event (i.e., unstable angina, myocardial infarction or coronary revascularization)
- A recent (30 days before randomization) active bleeding event
- Patients with known bleeding disorder (e.g., hemophilia)

### Randomization

- In case of emergency operation on the same day:
  - Randomization on the first postoperative day to ASA or placebo group
- In case of operation 24 h after last ASA intake:
  - Preoperative randomization to ASA or placebo group
- In case of elective surgery:
  - Preoperative randomization 5 days before surgery to ASA or Placebo group

### Surgical procedure

Burr-hole drainage and insertion of a drainage tube, by creating two burr holes in the skull, opening the dura mater, and relieving the fluid pressure caused by the chronic subdural hematoma. Eventually, a subdural (under the dura mater) or a subgaleal (over the skull bone) temporary drain is placed to continue draining the remaining fluid.

### Drug used

Aspirin cardio® (ASA), 100 mg 1–0-0/d, administered for 12 days after randomization.

### Control intervention

The placebo therapy was administered using a compound prepared by the Hospital Pharmacy of the University Hospital of Basel packed into similarly looking blisters.

### Study measurements

#### Preoperatively/at admission

- Cranial computed tomography (cCT): midline shift (MLS), hematoma width, hematoma characteristics (laminar/trabecular/septated/homogenous)
- Patient characteristics (age, medical history, oral anticoagulants/Clopidogrel/novel oral anticoagulants, history of falls, history of burr-hole drainage)
- Neurologic evaluation (NE), Glasgow Coma Scale (GCS), mRS, GOS, Markwalder score
- 12-channel electrocardiogram (ECG)
- Blood pressure (BP), saturation (SAT), heart rate (HR)
- Laboratory tests (hemoglobin, thrombocytes, quick, international normalized ratio (INR), platelet function assay bleeding time, troponin)

#### Intraoperatively

- Laboratory test 30 min to 1 h before surgery (troponin), if operation > 6 h after admission
- Amount of packed red blood cells given intraoperatively
- Intraoperative blood loss (in mL)

#### Postoperative Day 1

- NE, GCS, mRS, GOS, Markwalder score
- 12-channel ECG
- Vital parameters (BP, SAT, HR) four times daily
- Laboratory tests (hemoglobin, thrombocytes, quick, INR, troponin)
- cCT scan (MLS, hematoma width, recurrence)

#### Postoperative Day 2

- NE, GCS, mRS, GOS, Markwalder score
- 12-channel ECG
- Vital Parameters (BP, SAT, HR) four times daily
- Laboratory tests (hemoglobin, thrombocytes, quick, INR, troponin)
- Amount of fluid/blood collected in the drainage

#### Postoperative Day 7 or at discharge

- NE, GCS, mRS, GOS, Markwalder score
- Laboratory tests (hemoglobin, thrombocytes, quick, INR, troponin)
- 12-channel ECG
- Vital parameters (BP, SAT, HR)
- Hospitalization time
- Amount of packed blood cells given during hospitalization

#### Follow-up 1 (4–6 weeks postoperatively)

- NE, GCS, mRS, GOS, Markwalder score
- Vital parameters (BP, SAT, HR)
- cCT scan (MLS, hematoma width, recurrence)

#### Follow-up 2 (6 months postoperatively)

**Fig. 2:**
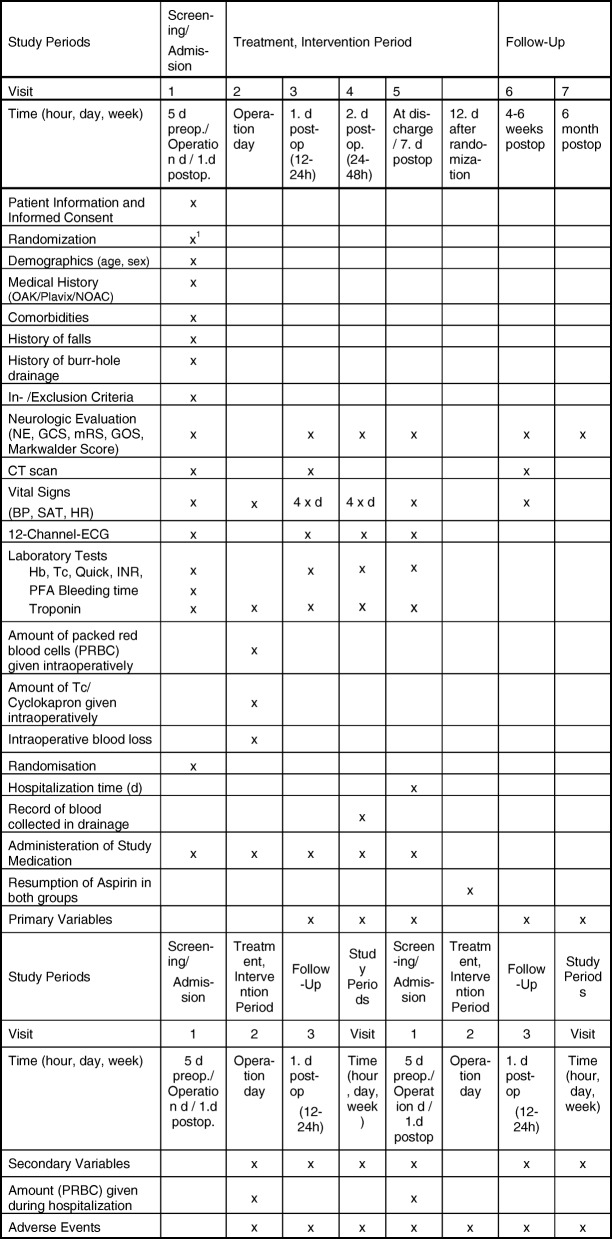
Study schedule

- NE, GCS, mRS, GOS, Markwalder score (Fig. 2)

### Statistical consideration

#### Number of participants with rationale

A systematic review by Almenawer et al. [23] reported an overall recurrence rate of approximately 10.7% for cSDH in the normal population. According to the literature, recurrence rates in the normal population range from 5% to 30% [24]. Recurrence rates have been described to reach 33% under antithrombotic therapy with ASA [12, 14]. In a retrospective study we conducted in patients where ASA treatment was continued, a recurrence rate of 22% was observed [15]. Based on the available data from the literature, we hypothesized that the recurrence rates after burr-hole drainage of cSDH under ASA treatment (π_ASA_) and without ASA treatment (under placebo, π_Placebo_) are 28% and 10%, respectively. The sample size was set to ensure at least 80% power (1 – β = 0.8), at a significance level of α = 5%. The sample size was estimated using a resampling procedure in which different sample sizes were each evaluated 999 times by drawing random samples of events based on these assumptions. This indicated that randomizing 142 patients should allow us to show the assumed difference in event rates between the groups.

The primary analysis will be performed on the full analysis set – consisting of all patients who are randomized to the study, regardless of protocol violations – and based on the intention to treat principle. The primary analysis is a comparison of incidence rate. The difference in incidence rate will be tested and a 95% confidence interval for the difference constructed. In case of many losses to follow-up, inverse probability censoring weights will be used to deal with missing outcome data. A sensitivity analysis will be performed on the per protocol analysis set. In the primary analyses, the type-I error rate for significance will be taken as α = 0.05. As secondary analysis, a time-to-event analysis will be performed comparing the hazard of recurrence between treatment arms using a Cox proportional hazards model. Secondary outcomes will be analyzed using generalized linear regression models with appropriate distribution family, depending on the outcome (binomial for binary outcome, Poisson for counts, Gaussian for continuous, normally distributed, outcomes).

#### Randomization

Each eligible patient will be allocated to ASA or placebo group either on the first postoperative day (in case of emergency operation on the same day), or preoperatively (in case of emergency operation 24 h after last ASA intake, or in case of elective surgery 5 days before operation).

Randomization will be performed by an independent individual using an unstratified block randomization procedure as implemented in the electronic data capture software SecuTrial. An allocation ratio of 1:1 and a block size of 8 will ensure a balance in sample size across both groups over time.

#### Methods of minimizing bias

Double-blind randomization in 1:1 allocation should reduce any bias due to patient or physician expectations. Randomization will be performed using a computer system (SecuTrial) through an independent individual. The medication will be numerically labelled at the Pharmacy of the University Hospital of Basel and will then be provided to the ward and applied to the patient. This will allow a double-blinded randomization (patient and treating physician will be blinded to the treatment). The placebo and ASA medications will be put into blisters so that they cannot be distinguished in their appearance. Data will be checked for protocol violation by the monitoring institution.

Throughout the trial, case report form (CRF) information will be inserted into a software especially designed for clinical trials (SecuTrial), through which mistakes are decreased to a minimum. At the end of the trial, the CRF data input will be verified by two individuals.

#### Blinding procedures

The study medication (ASA or placebo) will be provided as similar looking medication in blisters. The medication will be packed by the Pharmacy of the University Hospital of Basel and will be numerically labelled. The corresponding randomization list will be uploaded to the electronic data capture (EDC) system by the responsible Data Manager at the Clinical Trial Unit (CTU) Basel.

#### Unblinding procedures (Code break)

In case of problems that cannot be solved with ongoing randomization (safety concerns, withdrawal of participation, etc.), the participant’s allocated intervention will be revealed. Unblinding can be performed by authorized persons using the EDC system or by contacting the Pharmacy of the University Hospital of Basel. Each unblinding is documented and reported to the principal investigator. In case of a thrombotic event, ASA will be resumed regardless of the study arm in which the patient is allocated.

#### Compliance with study intervention

During hospitalization, compliance will be monitored by study nurses. After hospital discharge, patients will be instructed to continue taking medication until the 12th postoperative day. Compliance during the ambulatory phase of the trial will be controlled by patient or caregiver interviews during the clinical follow-up after 6 weeks. The patients will be explicitly asked if they took the study medication up to the 12th postoperative day and the empty blister and the rest of the medication, if there is one, will be collected. In case of non-compliance, a note-to-file will be completed and taken into account in the final statistical evaluation.

#### Data collection and follow-up for withdrawn participants

Withdrawn patients will be clinically followed up as non-participants, 4–6 weeks postoperatively in our outpatient clinic.

#### Trial-specific preventive measures

There are no specific restrictions associated with ASA/placebo medication. All of the patients included in the study are already under ASA treatment beforehand, therefore no restrictions apply.

#### Concomitant interventions (treatments)

Not applicable.

### Statistical analysis

#### Datasets to be analyzed, analysis populations

The full analysis set (FAS) will include all randomized patients and will be analyzed based on the intention to treat principle.

The per protocol set will include all patients of the FAS who had complete follow-up and who did not have serious protocol violations. Patients receiving the opposite treatment than that to which they were randomized will be excluded from this set.

#### Primary analysis

The difference in recurrence rate (π_Asp_–π_placebo_) will be tested by calculating a two-sided 95% confidence interval of the difference in proportions. Inverse probability censoring weights will be used to prevent selection bias in case of many losses to follow-up. The analysis will be performed primarily on the FAS. Sensitivity analyses will be performed on the per protocol set.

#### Secondary analyses

As a secondary analysis, a time-to-event approach will be taken to analyze the primary endpoint by using Cox-proportional hazards to estimate the difference in rate and time to recurrence. This can detect differences in time-to-event and incidence curves between the treatments, which could also be of relevance.

Other endpoints will be analyzed according to the type of variable, wherein binary endpoints will be analyzed using logistic regressions for the odds of occurrence and continuous endpoints (e.g., blood volume, operation time) will be analyzed using linear regression models if assumptions are not violated. For count data, Poisson or negative binomial models will be considered.

Subgroup analyses will be performed for male and female patients (e.g., patients with concomitant blood thinners). A test of interaction will be performed first, followed by an analysis within each subgroup.

Additional sub-analyses will be performed on patients taking Plavix/oral anticoagulants or novel oral anticoagulants to analyze if this therapy leads to an increased rate of bleeding.

#### Safety analysis

Summaries of adverse events by type and group will be prepared. Comparisons will be made between study arms for the number and types of adverse events.

#### Deviation from the original statistical plan

If substantial deviations of the analysis as outlined in this section are needed for whatever reason, the protocol will be amended. All deviations of the analysis from the protocol or from the detailed analysis plan will be listed and justified in a separate section of the final statistical report.

#### Handling of missing data and drop-outs

Missing data will be handled by the last observation performed. Sub-analysis will be performed on patients treated with concomitant blood thinners.

#### Monitoring

The electronic CRF (e-CRF) and source data will be reviewed for completeness and accuracy through regular monitoring provided by the study site. The study staff will be available for the monitoring visits in order to give access to the study files and provide any kind of support needed. The service will be provided by the Monitoring Department of CTU Basel.

#### Audits and inspections

Inspections by regulatory authorities during the study or following study completion are performed to ensure proper study conduct and data handling procedures according to International Conference on Harmonisation Good Clinical Practice guidelines and regulatory requirements. Inspections may include verification of all source documents, e-CRF, site files, and a visual inspection of the study site. Direct access to all documents and sites involved in the study will be provided by the study staff members. In case of an announced inspection, immediate notification of the other party is necessary.

#### Confidentiality, data protection

Direct access to source documents will be permitted for purposes of monitoring. The participants name or other personal identifiable data are not recorded in the CRF or the e-CRF. Subject confidentiality will be ensured by utilizing unique identification numbers for the corresponding data. Each participant will be coded by a number during the screening. After electronic enrolment to the study, the participant will be assigned with a unique personal study number (patient ID). All data collected for the study will be entered under the patient ID only.

The relation between the patient ID and participant’s name, address, date of birth and screening number will be documented in a written register (‘patient identification list’). The patient identification list will be stored in the research study office of the department of Neurosurgery, University Hospital of Basel. The Sponsor, Principal Investigator, Co-Investigator and study nurses will have access to the protocol and dataset, while the CTU statistician will have access to the statistical codes during and after the study.

Study data entered into the EDC system is only accessible by authorized persons. Once the data of all subjects is transferred to the EDC system, the database will be locked and closed for further data entry. The complete study dataset is exported, encrypted and transferred to the principal investigator through a secured channel by the responsible Data Manager at CTU Basel.

#### Follow-up of (serious) adverse events

Patients with reported serious adverse events during hospitalization will stay in hospital until the matter is resolved. If an intracranial bleeding has been diagnosed (and in some cases operated on), patients will be followed up in our outpatient clinic. NE (GCS, GOS, mRS and Markwalder score) as well as CT scan findings will be documented at 4–6 weeks and at 6 months postoperatively. Events other than those of a neurosurgical nature (thrombotic, etc.) will be followed up in a department in our hospital (e.g., cardiology) for as long as needed.

### Data entry/coding/security and storage

#### Case report forms

For each subject included in this study, a CRF will be completed, dated and signed by the study investigator. Each CRF will be kept current to reflect the participant status at each phase during the course of the study. All participants receive a unique identification number (patient ID) and no person-identifying data, such as name or initials, are collected in the CRF.

#### Specification of source documents

The investigator will maintain source documents for each patient included in the study, consisting of the paper CRF forms, case and visit notes (hospital or clinical medical records) containing demographic and medical information, laboratory data, electrocardiograms, and the results of any other tests or assessments.

#### Record keeping/archiving

All study data, including CRFs, Trial Master Files, and informed consent forms, will be archived for a minimum of 10 years after study termination or premature termination of the clinical trial. The study data will be archived in the research office of the Department of Neurosurgery University Hospital of Basel.

#### Data management

The study data recorded in the CRF will be transferred to a corresponding e-CRF by authorized persons (see Staff List).

#### Data management system

The e-CRF will be implemented by the responsible Data Manager at CTU Basel using the EDC software SecuTrial. The EDC software runs on a server maintained by the IT-department at University Hospital Basel.

#### Electronic and central data validation

Data entered into the e-CRF will be validated for completeness and discrepancies automatically. The data will be reviewed by the responsible investigator as well as by an independent monitor. The monitor will raise queries using the query management system implemented in SecuTrial. Designated investigators have to respond to the query and confirm or correct the corresponding data. Thereafter, the monitor can close the query.

#### Monitoring

The e-CRF and source data will be reviewed for completeness and accuracy through regular monitoring provided by the study site. The study staff will be available for the monitoring visits in order to allow access to the study files and provide any kind of support needed. The service will be provided by the Monitoring Department of CTU Basel.

#### Confidentiality, data protection

The Sponsor, Principal Investigator, Co-Investigator and study nurses will have access to the protocol and dataset. While the CTU statistician will have access to the statistical codes during and after the study.

Study data entered into the EDC system is only accessible by authorized persons. Once the data of all subjects is transferred to the EDC system, the database will be locked and closed for further data entry. The complete study dataset is exported, encrypted and transferred to the principal investigator through a secured channel by the responsible Data Manager at CTU Basel.

#### Ethical conduct of the study

The study will be performed in accordance to the protocol and with principles enunciated in the current version of the Declaration of Helsinki, the guidelines of Good Clinical Practice issued by the International Conference on Harmonisation and the Swiss Law and Swiss regulatory authority’s requirements.

#### Declaration of interest

There is no conflict of interest (independence, intellectual, financial, proprietary, etc.)

#### Patient information and informed consent

The investigators will explain to each participant the nature of the study, its purpose, the procedures involved, the expected duration, the potential risks and benefits, and any discomfort it may entail. All participants eligible for the study will be provided a participant information sheet and a consent form describing the study and providing sufficient information for them to make an informed decision about their participation in the study. The study population includes vulnerable participants. If patients are not capable of judgement (e.g., dementia/neurologic state) an informed consent of the next-of-keen and an independent physician will be obtained. If, in case of emergency, the next-of-keen is not available, informed consent will be obtained from an independent physician on site.

#### Participant privacy and confidentiality

The investigator affirms and upholds the principle of the participant’s right to privacy and that they shall comply with applicable privacy laws. Especially, anonymity of the participants shall be guaranteed when presenting the data at scientific meetings or publishing them in scientific journals.

Individual subject medical information obtained as a result of this study is considered confidential and disclosure to third parties is prohibited. Subject confidentiality will be further ensured by utilizing subject identification code numbers to correspond to treatment data in the computer files. For data verification purposes, authorized representatives of the Sponsor-Investigator, a competent authority (e.g., Swissmedic), or an ethics committee may require direct access to parts of the medical records relevant to the study, including participants’ medical history.

#### Early termination of the study

The Sponsor-Investigator may terminate the study prematurely according to certain circumstances, for example, ethical concerns, insufficient participant recruitment, when the safety of the participants is doubtful or at risk, alterations in accepted clinical practice that make the continuation of a clinical trial unwise, or early evidence of benefit or harm of the experimental intervention.

#### Protocol amendments

Substantial amendments are only implemented after the respective approvals of the competent ethics commitée (CEC) and competent authority (CA). Under emergency circumstances, deviations from the protocol to protect the rights, safety and well-being of human subjects may proceed without prior approval of the sponsor and the CEC/CA. Such deviations shall be documented and reported to the sponsor and the CEC/CA as soon as possible. All non-substantial amendments are communicated to the CA as soon as possible if applicable and to the CEC within the Annual Safety Report.

## Discussion

The continuation of ASA treatment during intracranial surgery might lead to perioperative bleeding complications such as higher perioperative blood loss, intracerebral bleeding, recurrent hematoma with or without neurologic deficits, or anemia. However, the risk of bleeding whilst operated under ASA is not well researched or documented in neurosurgical patients. On the other hand, discontinuation of ASA can lead to perioperative thromboembolic complications such as myocardial infarction, stroke, transient ischemic attack, PMI, embolic arterial occlusion of an extremity, or deep vein thrombosis. Data analyzing the rates of thromboembolic complications due to discontinuation of ASA treatment in the perioperative period are equally sparse.

To date, there is still no evidence-based consensus on how to treat patients undergoing burr-hole drainage of cSDH who are under ASA treatment. Therefore, this study will be of very high value for patients and physicians and will significantly improve our knowledge on the topic.

### Limitations

Although this is a randomized, double-blinded, placebo-controlled study, it has some limitations. Since burr-hole drainage is preformed either as an emergency or elective procedure, pre- and postoperative discontinuation time of ASA differs between the patients. By assuring that all patients under placebo will discontinue ASA at least 7 days after surgery, this bias should be minimal. Patients are often discharged before the study medication is sustained, and therefore compliance of the intake cannot be guaranteed in all patients. In order to minimize this bias, we will collect the empty medication boxes at the first follow-up visit. We expect the study to be multicentric by the first quartal of 2019, which will definitely lead to an improvement in the study quality.

#### Trial status

Version 2, 23.02.2017.

Recruitment beginning: 03/2018.

Recruitment end: 03/2022.

## Additional file

Additional file 1: SPIRIT 2013 Checklist: recommended items to address in a clinical trial protocol and related documents. (DOC 134 kb)

## Abbreviations

ASA: acetylsalicylic acid
BP: blood pressure
CA: competent authority
cCT: cranial computer tomography
CEC: competent ethics commitée
CTU: Clinical Trial Unit
CRF: case report form
cSDH: chronic subdural hematoma
ECG: electrocardiogram
e-CRF: electronic case report form
EDC: electronic data capture
FAS: full analysis set
GCS: Glasgow Coma Score
GOS: Glasgow Outcome Scale
HR: heart rate
INR: international normalized ratio
MLS: midline shift
mRS: modified Rankin scale
NE: neurologic evaluation
PMI: perioperative myocardial injury
SAT: oxygen saturation
